# Clinical Significance of Echocardiographic Parameters in Patients with Pulmonary Hypertension Associated with Interstitial Lung Disease

**DOI:** 10.3390/jcdd13080363

**Published:** 2026-08-01

**Authors:** Shingo Kato, Sho Kodama, Mai Azuma, Kazuki Fukui, Hideya Kitamura, Ryo Okuda, Tomohisa Baba, Minori Kinoshita, Tae Iwasawa, Shungo Sawamura, Naofumi Yasuda, Daisuke Utsunomiya, Takashi Ogura

**Affiliations:** 1 Department of Diagnostic Radiology, Yokohama City University, Yokohama 236-0004, Japanyasuda.nao.mz@yokohama-cu.ac.jp (N.Y.); d_utsuno@yokohama-cu.ac.jp (D.U.); 2 Department of Cardiology, Yokohama City University, Yokohama 236-0004, Japan; 3 Department of Cardiology, Kanagawa Cardiovascular Respiratory Center, Yokohama 236-0051, Japan; 4 Department of Respiratory Medicine, Kanagawa Cardiovascular Respiratory Center, Yokohama 236-0051, Japan; 5 Department of Diagnostic Radiology, Kanagawa Cardiovascular Respiratory Center, Yokohama 236-0051, Japan

**Keywords:** interstitial lung disease, pulmonary hypertension, echocardiography, left ventricular ejection fraction, prognosis

## Abstract

Background: Pulmonary hypertension (PH)-complicating interstitial lung disease (ILD) is a devastating condition that severely limits exercise capacity, diminishes quality of life (QOL), and ultimately determines survival. The pathophysiological assessment of ILD-PH has traditionally focused on right ventricular (RV) parameters reflecting RV pressure overload and dysfunction. However, under extreme RV pressure overload where blood supply to the left heart is severely restricted, the prognostic role of left ventricular (LV) function—which is ultimately responsible for maintaining systemic cardiac output—remains poorly understood. This study aimed to comprehensively evaluate the hemodynamics of ILD patients using non-invasive transthoracic echocardiography, to determine the prognostic importance of LV functional parameters, particularly left ventricular ejection fraction (LVEF). Methods: This single-center, retrospective, observational study included 415 ILD patients diagnosed and treated at the Kanagawa Cardiovascular Respiratory Center. All patients underwent transthoracic echocardiography at diagnosis, from which parameters such as estimated right ventricular systolic pressure (RVSP), tissue Doppler-derived Average *e’*, and LVEF were obtained. A multivariable Cox proportional hazards model was applied to evaluate the association between these echocardiographic parameters and all-cause mortality. To account for the prognostic impact of the underlying disease, we conducted a stratified analysis of idiopathic pulmonary fibrosis (IPF) and non-IPF cohorts. Furthermore, to evaluate LV function under the most severe hemodynamic compromise, a subgroup analysis was restricted to IPF patients with elevated RVSP (≥median). Results: During a median follow-up of 27.2 months, 75 (18.1%) of the 415 patients died. In the multivariable Cox analysis of the overall cohort, a decreased Average *e’* (HR 0.861, *p* = 0.0038) emerged as a strong independent predictor of poor prognosis. Stratified analysis revealed that in the non-IPF group (*n* = 208, 15 events), none of the variables achieved statistical significance. Conversely, in the IPF group (N = 207, 60 events), both LV diastolic function (Average *e’*, *p* = 0.0199) and LV systolic function (LVEF, HR 0.969, 95% CI 0.940–0.998, *p* = 0.0305) were extracted as significant prognostic predictors. Most notably, in the stepwise multivariable model restricted to the “IPF with high RVSP” subgroup (N = 104), the prognostic significance of diastolic function (Average *e’*) was lost (*p* = 0.4577), whereas LVEF (HR 0.965, 95% CI 0.936–0.995, *p* = 0.0229) emerged as the sole independent predictor of mortality. Conclusions: In the overall ILD-PH cohort, reduced LV diastolic function (Average *e’*) is a strong independent predictor of mortality. While elevated RVSP and low BMI showed a trend toward worsening prognosis, they were not statistically significant in multivariable analysis. The prognostic contribution of LV function is primarily driven by the IPF patient group, which has an inherently poor prognosis. Furthermore, in the severe subgroup of IPF patients heavily burdened by right heart overload, LV pump function (LVEF) becomes an independent, critical determinant of ultimate survival. Therefore, routine measurement and careful monitoring of the universal parameter LVEF are of paramount importance for the risk stratification of high-risk patients under such complex hemodynamics.

## 1. Introduction

Interstitial lung disease (ILD) comprises a group of intractable respiratory disorders characterized by chronic inflammation and progressive fibrosis of the alveolar walls, frequently complicated by pulmonary hypertension (PH) as the disease progresses [1,2,3]. The prevalence of ILD-PH, which belongs to Group 3 PH in the World Health Organization (WHO) classification [4,5], varies widely depending on the underlying disease subtype and screening methods, but is estimated to reach 30% to 50% in advanced idiopathic pulmonary fibrosis (IPF). The presence of PH causes a marked decline in exercise capacity, exacerbation of hypoxemia, and induction of irreversible right heart failure, independently and significantly worsening the prognosis of ILD patients, as clearly demonstrated by numerous previous cohort studies [6,7,8,9].

Currently, right heart catheterization (RHC) is the indispensable gold standard for the definitive diagnosis of PH and the acquisition of detailed hemodynamic parameters (mean pulmonary arterial pressure, pulmonary vascular resistance, pulmonary capillary wedge pressure, etc.) [7,10]. However, RHC is an invasive procedure, making it extremely difficult from the perspective of physical burden to repeatedly perform as a routine screening test for severe ILD patients who suffer from severe respiratory dysfunction and hypoxemia. Therefore, in routine clinical practice, non-invasive and repeatable transthoracic echocardiography is positioned as the most important first-line modality for the early detection, disease monitoring, and risk stratification of PH in ILD patients [11,12,13].

Echocardiography offers the significant advantage of comprehensively evaluating cardiopulmonary hemodynamics in a single examination. It allows not only the simple estimation of right ventricular systolic pressure (RVSP) using tricuspid regurgitation velocity (TRV), but also the assessment of RV performance indices, including the Tei Index (myocardial performance index), Tricuspid Annular Plane Systolic Excursion (TAPSE), and Right Ventricular Fractional Area Change (RVFAC), and left ventricular morphology and function (LVEF, E/*e’*, Average *e’*) [14,15,16]. To date, right ventricular dysfunction and elevated RVSP have been strongly associated with a poor prognosis not only in connective tissue disease-associated ILD (CTD-ILD) like systemic sclerosis, but also in IPF [17,18,19]. However, in the highly heterogeneous and complex population of ILD-PH, there is still insufficient consensus on which of the myriad echocardiographic parameters serves as a truly independent and universal prognostic predictor [20,21].

**Figure 1:**
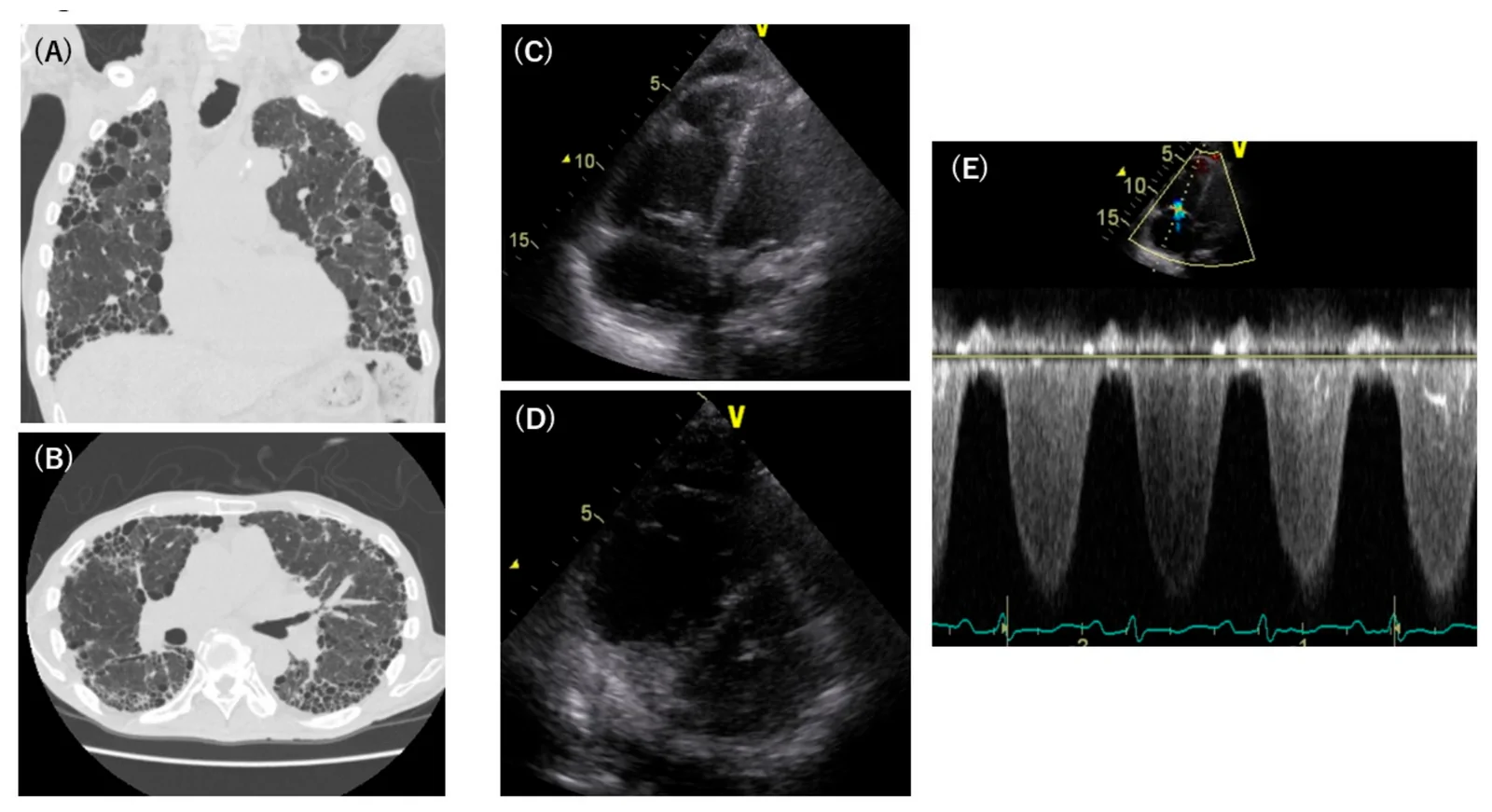
Representative chest HRCT and echocardiographic images of a patient with severe interstitial lung disease and pulmonary hypertension (ILD-PH). (**A**) Coronal and (**B**) axial views of chest HRCT demonstrating extensive fibrotic changes and honeycombing predominantly in the lower lobes. (**C**) Apical four-chamber view on transthoracic echocardiography showing severe right ventricular dilation and a leftward shift in the interventricular septum, indicating right heart volume and pressure overload. (**D**) Parasternal short-axis view at the papillary muscle level revealing a D-shaped left ventricle, consistent with severe right ventricular pressure overload (ventricular interdependence). (**E**) Continuous-wave Doppler of the tricuspid regurgitation jet showing a high-velocity signal (TR Vmax = 3.6 m/s, yielding a pressure gradient of 52 mmHg), used to estimate the markedly elevated right ventricular systolic pressure (RVSP).

Recently, particular attention has been paid to the direct impact of excessive right heart overload on left ventricular function via ventricular interdependence. When right ventricular pressure overload increases, the interventricular septum is displaced toward the left ventricle (D-shape), physically reducing LV diastolic volume and compliance. Concurrently, the impairment of the pulmonary circulation reduces the actual preload returning to the left atrium and ventricle, causing “underfilling” [14,21,22] (Figure 1). In this state of extreme preload reduction, the actual pump function of the left ventricle—the left ventricular ejection fraction (LVEF)—is thought to play a decisive role in maintaining sufficient oxygen delivery and cardiac output to the body. However, because evaluations in ILD patients have long focused primarily on right heart function, few studies have rigorously investigated whether the simple, classic parameter LVEF serves as a truly independent prognostic predictor in advanced PH cases.

In this study, we aimed to:

1. Comprehensively analyze the association between various echocardiographic parameters (both right and left heart indices) and all-cause mortality in a large cohort of patients with diverse ILD treated at our center.
2. Identify the specific echocardiographic parameters that independently determine prognosis in the overall cohort and across different ILD subtypes (IPF vs. non-IPF).
3. Verify, through a subgroup analysis of patients with advanced right ventricular overload (high RVSP), whether LVEF measurement serves as a critical, final risk-stratification determinant in routine clinical practice under complex hemodynamics.

## 2. Methods

### 2.1. Study Population and Design

**Figure 2:**
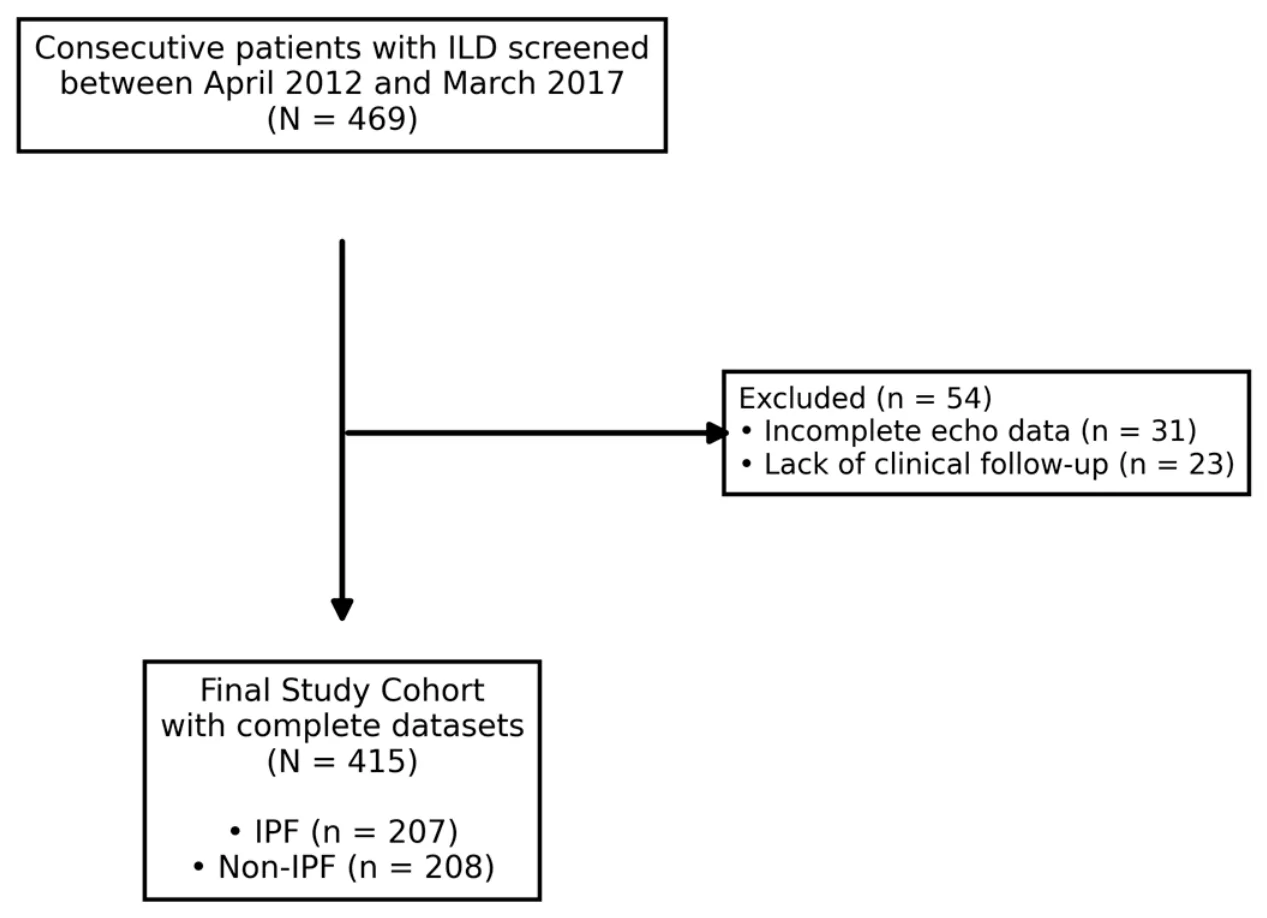
Study Flowchart. Flowchart displaying the screening, exclusion criteria, and final baseline enrollment of the ILD cohort (*n* = 415). Out of 469 screened patients, 54 were excluded due to incomplete echocardiographic records or lack of clinical follow-up, resulting in the final cohort of 415 patients.

This single-center, retrospective observational study screened consecutive ILD patients who were diagnosed and treated at the Kanagawa Cardiovascular Respiratory Center (KCRC), a specialized tertiary referral facility for interstitial lung diseases, between April 2012 and March 2017. The patient selection process is illustrated in the study flowchart (Figure 2). Out of 469 patients initially evaluated, 54 patients were excluded due to incomplete echocardiographic data or lack of clinical follow-up, resulting in a final study cohort of 415 patients with complete datasets. All included patients underwent comprehensive baseline clinical, laboratory, and transthoracic echocardiographic evaluations.

The study population encompassed a broad range of ILD subtypes, which were classified into two primary categories: idiopathic pulmonary fibrosis (IPF) and Non-IPF. The diagnosis of all ILD subtypes was strictly established in accordance with international consensus guidelines through Multidisciplinary Discussion (MDD) involving expert pulmonologists, thoracic radiologists, and pathologists. Specifically, IPF was diagnosed based on the joint guidelines of the American Thoracic Society (ATS), European Respiratory Society (ERS), Japanese Respiratory Society (JRS), and Latin American Thoracic Association (ALAT). Nonspecific interstitial pneumonia (NSIP) was diagnosed via histopathological confirmation from surgical lung biopsy or characteristic high-resolution computed tomography (HRCT) findings. Connective tissue disease-associated interstitial lung disease (CTD-ILD) was diagnosed in collaboration with rheumatologists, based on the established classification criteria of the American College of Rheumatology (ACR) and European Alliance of Associations for Rheumatology (EULAR) for systemic sclerosis, rheumatoid arthritis, and dermatomyositis/polymyositis. Other Non-IPF etiologies included chronic hypersensitivity pneumonitis and cryptogenic organizing pneumonia. The primary clinical endpoint was all-cause mortality during the follow-up period, verified through medical records or official referral documentation.

### 2.2. Transthoracic Echocardiographic Measurements

Standard transthoracic echocardiography was performed on all patients at the time of diagnosis by experienced, board-certified sonographers and cardiologists using high-end ultrasound systems. All measurements were conducted in accordance with the standard guidelines of the Japanese Society of Echocardiography and the American Society of Echocardiography (ASE) [14,15,16]. Representative chest HRCT and echocardiographic images are shown in Figure 2.

#### 2.2.1. Right Ventricular Function and Pulmonary Artery Pressure Indices

As a non-invasive surrogate marker of pulmonary artery pressure, the maximum velocity of tricuspid regurgitation (TR Vmax or TRV) was measured using continuous-wave Doppler. The tricuspid regurgitation pressure gradient (TRPG) was calculated using the simplified Bernoulli equation (4 × TRV^2^). Right ventricular systolic pressure (RVSP) was then estimated as RVSP = TRPG + RAP, where right atrial pressure (RAP) was estimated based on the inferior vena cava (IVC) diameter and its respiratory variation (collapsibility index). Specifically, IVC diameter was measured from the subcostal view at end-expiration (maximum diameter) and end-inspiration (minimum diameter), 1.0–2.0 cm from the junction with the right atrium. The collapsibility index was calculated as (maximum diameter − minimum diameter)/maximum diameter. RAP was assigned as 3 mmHg (IVC diameter < 2.1 cm and collapsibility > 50%), 15 mmHg (IVC diameter > 2.1 cm and collapsibility < 50%), or 8 mmHg (other intermediate scenarios) [14,16]. To avoid potential bias associated with RAP estimation, TRV alone was also recorded and analyzed for clinical concordance. The RV Tei Index (myocardial performance index), reflecting global RV performance, was calculated as (IVCT + IVRT)/ET (where IVCT is isovolumetric contraction time, IVRT is isovolumetric relaxation time, and ET is ejection time), obtained via pulsed-wave Doppler at the lateral tricuspid annulus or continuous-wave Doppler of the RV outflow. Tricuspid annular plane systolic excursion (TAPSE) was measured using M-mode at the lateral tricuspid annulus. Right ventricular fractional area change (RVFAC) was calculated as (RVEDA − RVESA)/RVEDA × 100 (%), where RVEDA and RVESA represent RV end-diastolic and end-systolic areas, respectively.

#### 2.2.2. Left Ventricular Morphology and Function Indices

For LV morphological parameters, interventricular septal thickness in diastole and systole (IVSd, IVSs) and LV posterior wall thickness (LVPWd) were measured using M-mode or 2D imaging in the parasternal long-axis view. Systolic function was evaluated by left ventricular ejection fraction (LVEF), measured using the biplane Simpson’s method (biplane method of disks) in apical four- and two-chamber views. In cases with poor acoustic windows where endocardial border delineation was suboptimal, LVEF was estimated using the Teichholz method based on end-diastolic and end-systolic internal dimensions (LVIDd, LVIDs). The biplane Simpson’s method was the primary approach utilized for LVEF measurement (applied in approximately 95% of patients), whereas the Teichholz method was strictly reserved as a fallback option only in the remaining 5% of cases where poor acoustic windows due to advanced lung hyperinflation or emphysema prevented clear endocardial border delineation. Mitral inflow velocities (E and A waves) were measured using pulsed-wave Doppler at the mitral leaflet tips to assess diastolic function. Tissue Doppler imaging (TDI) was utilized to measure mitral annular velocities at the septal and lateral borders. Average *e’* (early diastolic mitral annular velocity) was calculated as the mean of the septal and lateral *e’* velocities. Average E/*e’* ratio was calculated as mitral E wave velocity divided by Average *e’* to estimate LV filling pressure. The LV eccentricity index, indicating ventricular interdependence, was measured at end-diastole in the parasternal short-axis view at the papillary muscle level.

### 2.3. Statistical Analysis

Continuous variables were expressed as mean ± standard deviation or median (interquartile range) depending on their distribution, and categorical variables were presented as frequencies and percentages (%). Patient survival time was analyzed using the Kaplan–Meier method to plot survival curves, and group comparisons were performed using the Log-rank test.

To quantitatively evaluate the impact of clinical parameters (age, sex, BMI, etc.) and echocardiographic indices on prognosis (all-cause mortality), we first performed univariate and multivariable Cox proportional hazards analyses on the overall cohort (N = 415), calculating hazard ratios (HR) and 95% confidence intervals (CI).

Furthermore, considering the impact of the underlying disease on prognosis, we conducted a “stratified analysis” dividing the ILD subtypes into two groups: idiopathic pulmonary fibrosis (IPF)—the subtype with the worst prognosis—and Non-IPF. Finally, to verify one of the primary objectives of this study—“the significance of left ventricular function under extreme conditions of advanced right heart overload”—we conducted a subgroup analysis restricted to IPF patients with an RVSP at or above the median (“IPF and High RVSP group”). In this subgroup, a multivariable Cox analysis model adjusted for fundamental risk factors (age, sex, BMI, RVSP) was constructed to strictly evaluate whether various LV parameters (LVEF, Average *e’*, etc.) remained independent prognostic factors.

All statistical analyses were performed using Python version 3.11 (64-bit; Python Software Foundation, Wilmington, DE, USA), and a *p*-value < 0.05 was considered statistically significant. This study was conducted with the rigorous review and approval of the Institutional Review Board of the Kanagawa Cardiovascular Respiratory Center (Approval number: KCRC-26-0003), and patients were guaranteed ample opportunity to refuse participation via an opt-out method.

## 3. Results

### 3.1. Patient Characteristics and Univariate Cox Proportional Hazards Analysis in the Overall Cohort (N = 415)

The baseline clinical characteristics of the study population are summarized in Table 1. The overall cohort (N = 415) had a mean age of 69.3 ± 9.4 years, included 250 (60.2%) males, and had a mean BMI of 23.0 ± 3.8 kg/m^2^. Patients with a primary diagnosis of idiopathic pulmonary fibrosis (IPF) accounted for 207 cases (49.9%). Baseline hemodynamic parameters obtained via echocardiography revealed a mean estimated right ventricular systolic pressure (RVSP) of 36.72 ± 12.61 mmHg, a mean left ventricular ejection fraction (LVEF) of 64.73 ± 7.13%, and a mean Average *e’* (an index of left ventricular diastolic function) of 7.11 ± 1.91 cm/s.

During a median follow-up of 27.2 months (interquartile range [IQR] 13.6–45.1 months), 75 (18.1%) mortality events were confirmed among the 415 ILD patients. These were primarily due to acute exacerbation of the underlying disease, progression of chronic respiratory failure, and circulatory collapse secondary to right heart failure.

Univariate Cox proportional hazards analysis evaluated the impact of clinical background factors and individual echocardiographic parameters on prognosis.

Among clinical backgrounds, increased age (HR 1.052, 95% CI 1.022–1.082, *p* = 0.0005) and decreased BMI (HR 0.929, 95% CI 0.872–0.989, *p* = 0.0217) were highly significant poor prognostic factors. In contrast, sex, comorbidities such as diabetes and hypertension, and differences in underlying disease (IPF vs. non-IPF) showed no significant association with the risk of all-cause mortality in this cohort.

Focusing on echocardiographic parameters, continuous indices indicating right heart overload and dysfunction emerged as significant prognostic factors in the overall cohort. Specifically, elevated RVSP (HR 1.017, 95% CI 1.005–1.029, *p* = 0.0064) and high RV TEI Index (HR 4.835, 95% CI 1.576–14.840, *p* = 0.0059) significantly increased the risk of mortality. Furthermore, parameters related to left heart function also yielded significant results: decreased LVEF indicating reduced LV systolic function (HR 0.963, 95% CI 0.932–0.995, *p* = 0.0240), decreased Average *e’* indicating reduced LV diastolic function (HR 0.822, 95% CI 0.726–0.931, *p* = 0.0021), and increased A wave (HR 1.013, 95% CI 1.003–1.024, *p* = 0.0145) were all identified as significant predictors of a poor prognosis.

To address specific clinically relevant right ventricular function thresholds (requested by the Academic Editor), we performed univariate Cox analyses using categorical cut-offs. An estimated RVSP of ≥50 mmHg was associated with a significant increase in mortality in both the overall cohort (HR 2.157, 95% CI 1.295–3.592, *p* = 0.0032) and the IPF subgroup (HR 1.879, 95% CI 1.052–3.358, *p* = 0.0332). Conversely, a reduced TAPSE of <15 mm showed a trend towards worsening survival but did not achieve statistical significance in either the overall cohort (HR 1.446, 95% CI 0.725–2.884, *p* = 0.2955) or the IPF subgroup (HR 1.435, 95% CI 0.683–3.015, *p* = 0.3407).

### 3.2. Multivariable Cox Proportional Hazards Analysis in the Overall Cohort (N = 415)

To statistically adjust for the complex confounders between the parameters observed in the univariate analysis and to identify the true independent factors determining prognosis, a multivariable Cox proportional hazards analysis was performed (Table 2). The core variables incorporated into the model were age, sex, BMI, RVSP, Average *e’*, and LVEF.

The analysis revealed that in the overall cohort, a decrease in Average *e’* (HR 0.861, 95% CI 0.778–0.953, *p* = 0.0038), an index of left ventricular diastolic function, was identified as an extremely powerful and independent prognostic predictor. Additionally, increased age (HR 1.025, 95% CI 1.004–1.047, *p* = 0.0225) and male sex (HR 1.652, 95% CI 1.117–2.445, *p* = 0.0123) were also significant poor prognostic factors. Meanwhile, decreased BMI (HR 0.950, 95% CI 0.903–1.000, *p* = 0.0504) and elevated RVSP (HR 1.013, 95% CI 1.000–1.026, *p* = 0.0515) exhibited only a borderline trend towards significance.

### 3.3. Stratified Analysis (IPF vs. Non-IPF): Contribution of Left Ventricular Function to Prognosis

Next, to examine how the strong prognostic predictive ability of left heart function (Average *e’*) in the overall cohort is modified by differences in underlying diseases, we conducted a stratified multivariable Cox analysis between the IPF group (N = 207) and the Non-IPF group (N = 208) (Table 3).

**Figure 3:**
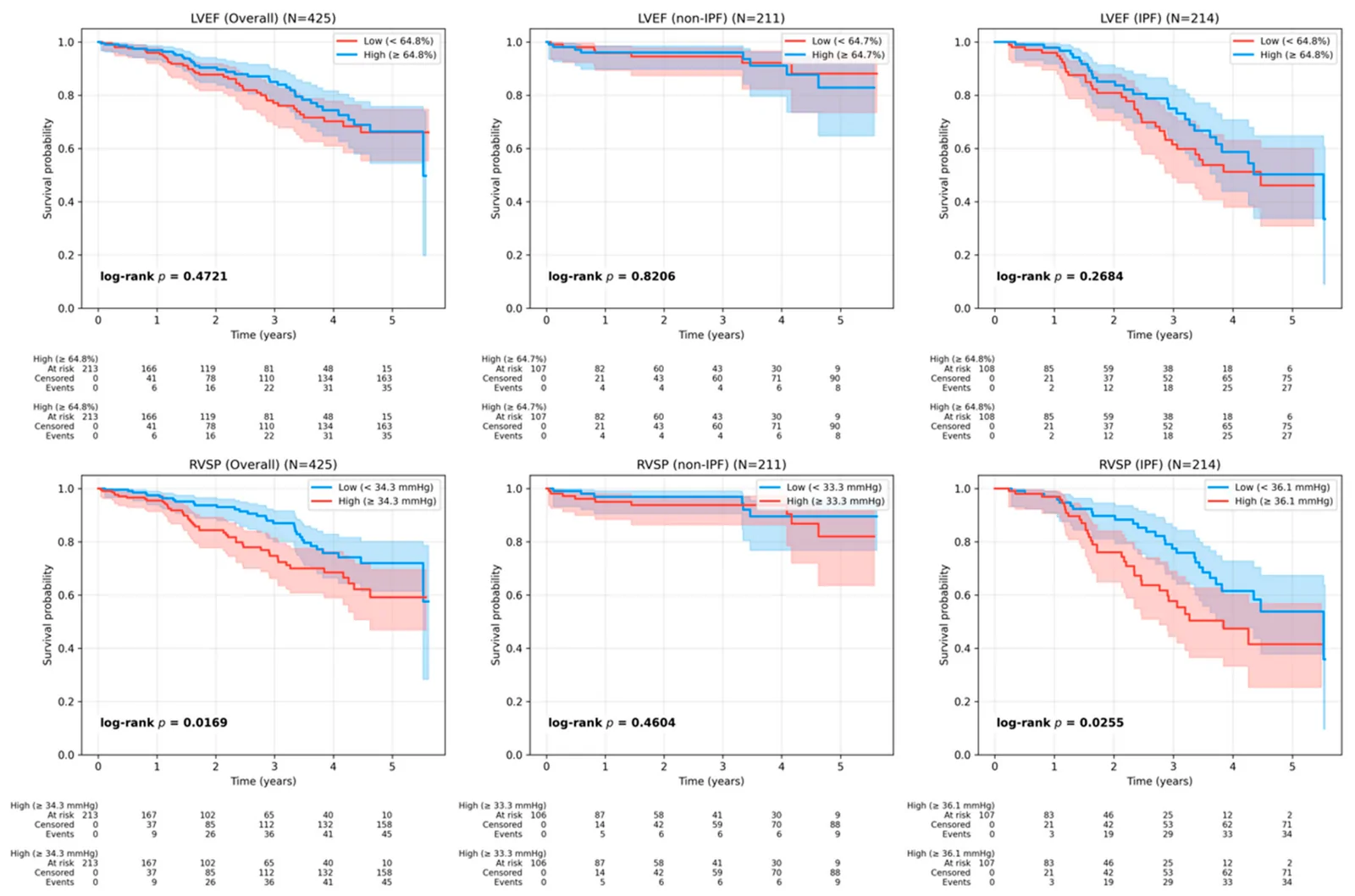
Kaplan–Meier survival curves stratified by Left Ventricular Ejection Fraction (LVEF) and Right Ventricular Systolic Pressure (RVSP) across the overall, non-IPF, and IPF cohorts. The panels display survival curves demonstrating the prognostic impact of LVEF and RVSP in different interstitial lung disease (ILD) subtypes. While RVSP shows varying degrees of prognostic significance, decreased LVEF emerges as a significant predictor of all-cause mortality, particularly in the IPF subgroup.

Strikingly, in the Non-IPF group (15 events), none of the variables held statistical significance. In stark contrast, in the IPF group (60 events)—which has an exceptionally poor prognosis—not only Average *e’*, reflecting diastolic function (HR 0.854, 95% CI 0.748–0.974, *p* = 0.0199), but also LVEF, reflecting systolic function (HR 0.969, 95% CI 0.940–0.998, *p* = 0.0305), clearly emerged as independent prognostic predictors (Figure 3). This demonstrated that the dynamic phenomenon wherein “declining left ventricular function worsens prognosis” was primarily driven by the IPF group.

### 3.4. Subgroup Analysis of IPF Patients with High RVSP

To identify the impact of left ventricular function on ultimate prognosis in the extreme patient subgroup where ILD-PH pathology has progressed and right heart overload is severe, we restricted our multivariable Cox analysis exclusively to the “IPF and high RVSP group” (N = 104, 33 events), comprising IPF patients with an RVSP above the median of 35.7 mmHg (Table 4).

**Figure 4:**
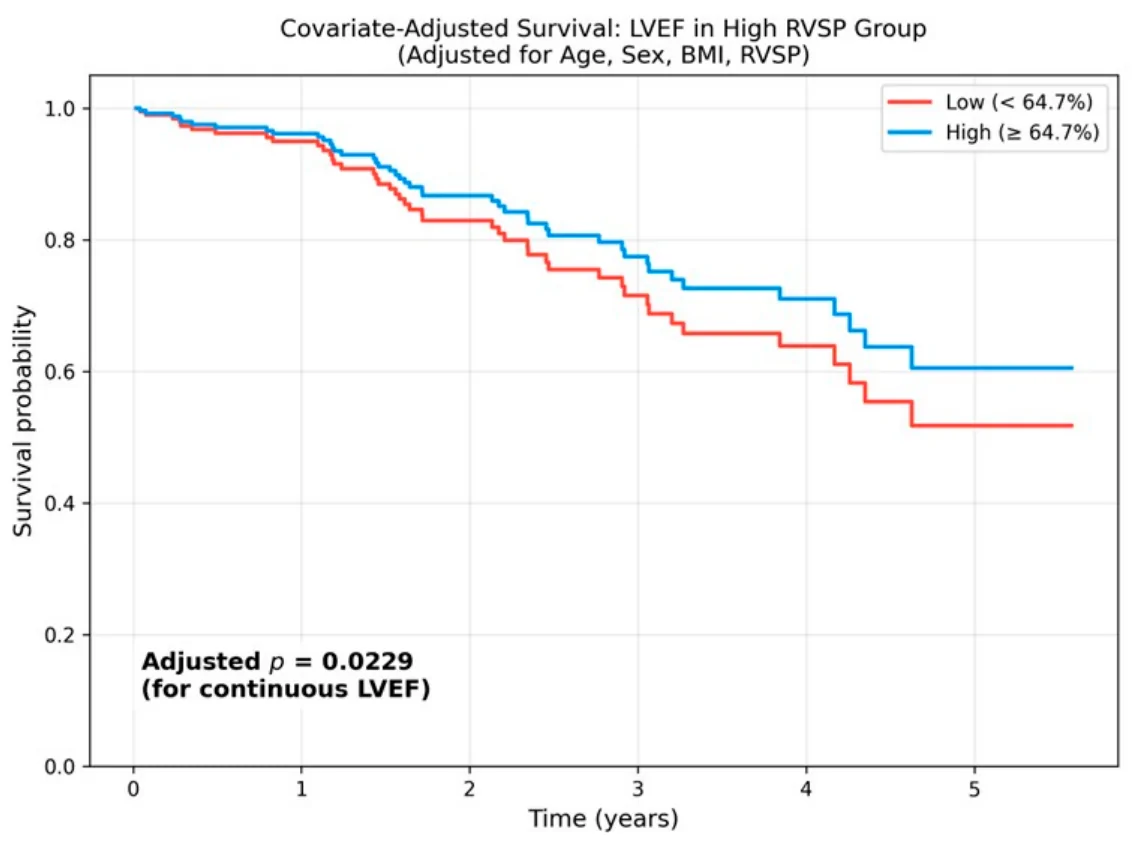
Covariate-adjusted survival curve stratified by Left Ventricular Ejection Fraction (LVEF) in the high-RVSP patient group. The survival curves were adjusted for age, sex, body mass index, and continuous RVSP using a multivariable Cox proportional hazards model. In this subgroup of patients with elevated right ventricular pressure overload (RVSP ≥ median), lower baseline LVEF is significantly and independently associated with poorer survival, highlighting the critical role of left ventricular systolic reserve in severe disease states.

Left ventricular diastolic function (Average *e’*), which had been a robust prognostic factor in the overall cohort and the overall IPF group, entirely lost its significance under these harsh hemodynamic conditions (HR 0.932, 95% CI 0.775–1.122, *p* = 0.4577). In stark contrast, LVEF—an index of left ventricular systolic function—was the sole parameter to emerge as an independent prognostic predictor (HR 0.965, 95% CI 0.936–0.995, *p* = 0.0229) (Figure 4), overriding all other confounding factors including age, sex, BMI, and RVSP.

## 4. Discussion

This study comprehensively evaluated the prognostic predictive capacity of various parameters derived from non-invasive transthoracic echocardiography in patients with ILD-PH, a population characterized by an exceptionally poor prognosis and formidable clinical management challenges. As a result, in the overall cohort (N = 415), left ventricular diastolic function (Average *e’*) emerged as a potent independent predictor of mortality. However, a series of stepwise analyses yielded highly suggestive clinical insights. Namely, the phenomenon wherein left ventricular function dictates prognosis is predominantly driven by “IPF patients.” Furthermore, within the severe context of “IPF coupled with high RVSP” where right heart overload is advanced, the impact of diastolic function dissipates, leaving the “intrinsic systolic pump function of the left ventricle (LVEF)” as the sole determinant of patient survival. Based on these findings, we elaborate on the following principal perspectives.

### 4.1. Significance and Limitations of Right Heart Overload Indices in the Overall ILD-PH Cohort

In our overall cohort, although an elevation in RVSP was a significant factor for worsening prognosis in the univariate analysis, it showed only borderline significance (*p* = 0.0515) in the multivariable analysis and did not solidify as a clear independent prognostic predictor. The progression of PH in ILD patients entails more than just hypoxic pulmonary vasoconstriction (HPV); it is a complex interplay involving the irreversible physical destruction of the capillary bed due to extensive lung fibrosis, endothelial dysfunction, thrombosis in situ, and vascular remodeling [10,12,23]. This persistent and progressive escalation in afterload easily surpasses the limits of right ventricular compensatory mechanisms (e.g., wall hypertrophy) and ultimately culminates in irreversible right heart failure [7,11,24].

Recent studies have demonstrated that, rather than RVSP alone, mild elevations in pulmonary vascular resistance (PVR), mean pulmonary arterial pressure (mPAP), and indices of right ventricular functional and volume overload more strongly dictate the prognosis of ILD patients [25,26]. Notably, Sato et al. proved in a large cohort that a mild elevation in PVR acts as an independent mortality predictor regardless of the mPAP value [25]. The current study extends these findings by demonstrating that a categorical threshold of RVSP ≥ 50 mmHg is a highly significant predictor of mortality (HR 2.157 in the overall cohort, and HR 1.879 in the IPF cohort), highlighting that patients exceeding this threshold constitute a particularly high-risk subgroup. Conversely, categorical TAPSE (<15 mm) was not a statistically significant predictor in this cohort, which may reflect the relative preservation of longitudinal RV function in early-to-moderate stages of Group 3 PH, or the higher sensitivity of pressure-derived markers over load-dependent longitudinal indices under these specific conditions. The loss of significance of RVSP—a non-invasive surrogate—in our multivariable analysis may be attributable to the fact that a simple pressure estimate (RVSP) cannot fully capture the intricate processes of right heart failure, such as the actual elevation in PVR and the consequent functional and volumetric burden on the right ventricle.

### 4.2. Ventricular Interdependence and the Dynamic Clinical Significance of Left Heart Function (Average e’ and LVEF)

The most novel and vital finding derived from this study is the dynamic relationship between right heart overload and left heart function (particularly LVEF), explained through the concept of ventricular interdependence.

In this study, a decline in Average *e’*, an index of LV diastolic function, was extracted as an extremely powerful prognostic exacerbating factor in the multivariable analysis of the overall cohort (*p* = 0.0038). Elderly ILD patients frequently possess underlying myocardial stiffness (HFpEF-like elements), and a decline in diastolic function can be said to acutely reflect disease progression [27]. Next, stratified analysis by IPF and non-IPF groups revealed that the phenomenon wherein LVEF and *e’* dictate prognosis was observed exclusively in the IPF patient group, which accounts for the vast majority of mortality events and exhibits rapid disease progression [28].

However, it is most noteworthy that in the subgroup analysis of the “IPF and high RVSP group” (N = 104), where right ventricular pressure overload had highly progressed, the significance of Average *e’* entirely vanished. Instead, “LVEF”—an index of LV systolic function—distinctly surfaced as the sole, strongest independent prognostic predictor (HR 0.965, *p* = 0.0229).

**Figure 5:**
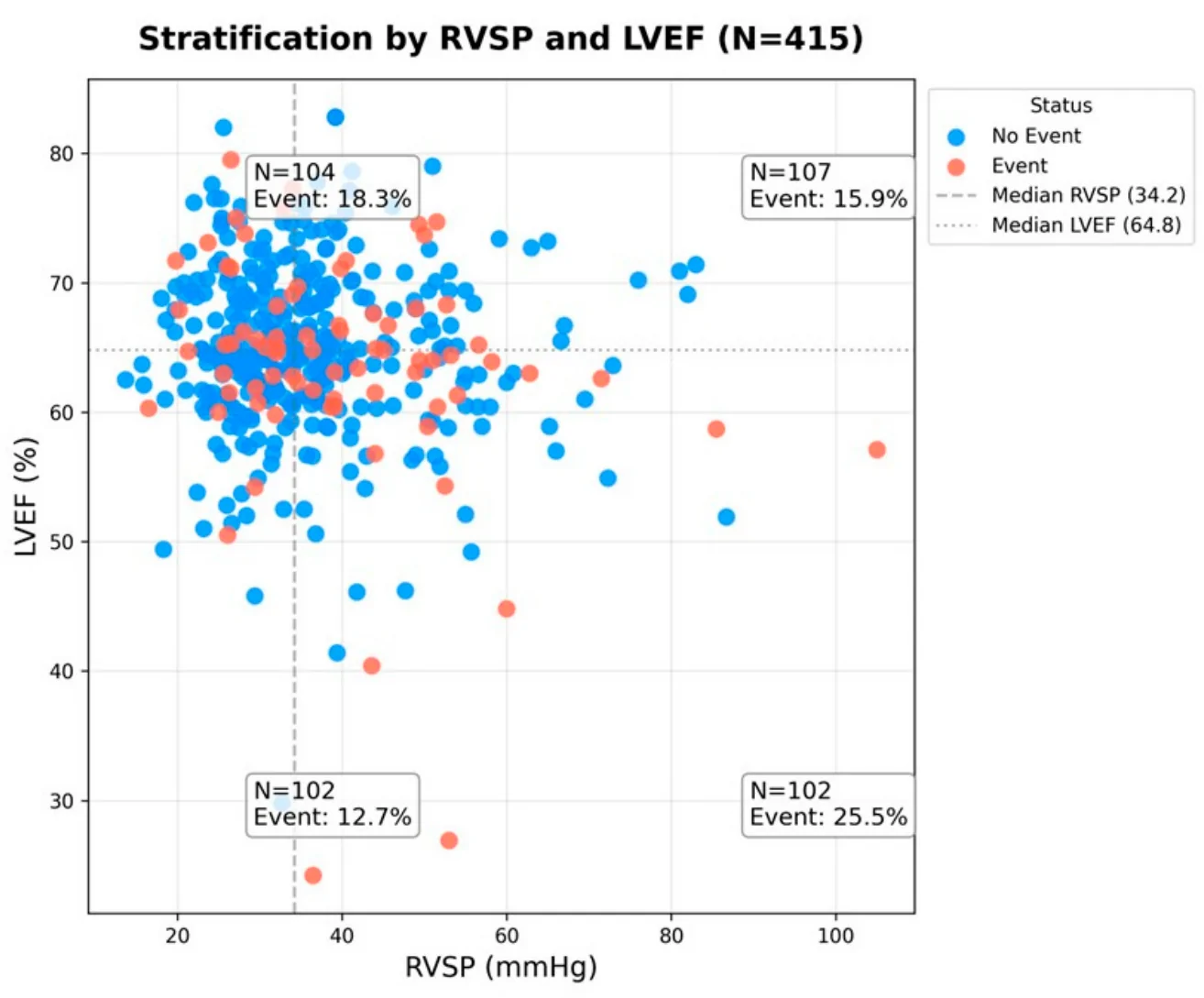
Scatter plot demonstrating the distribution of ILD patients according to RVSP and LVEF (N = 415). The plot illustrates the complex relationship between right ventricular pressure overload and the consequent impairment of left ventricular systolic function via ventricular interdependence.

In Group 3 PH, as right ventricular pressure and volume overload increase, the interventricular septum is physically displaced toward the left ventricle (forming a D-shape), thereby mechanically restricting the left ventricular diastolic volume and compliance. This is known as “ventricular interdependence,” occurring because the two ventricles share the confined space of the pericardium [14,22] (Figure 5). Furthermore, the destruction of the pulmonary vascular bed drastically reduces the volume of blood returning from the lungs to the left atrium and ventricle (underfilling) [29]. In this state of profoundly diminished preload, the cardiac output required to sustain life becomes heavily reliant on the “intrinsic systolic force of the left ventricle (LVEF).” While a decline in diastolic function reflected disease progression in the overall cohort, under conditions where right heart overload has advanced and hemodynamics are constrained, the more direct determinant of ultimate survival becomes whether the heart can “pump sufficient blood to the systemic circulation” (i.e., whether LVEF is maintained).

At the individual level, clinicians might question the utility of parameters with hazard ratios close to unity (e.g., LVEF HR of 0.965 in the high-RVSP IPF subgroup). However, it is essential to emphasize that these hazard ratios are calculated per unit increase (i.e., per 1% of LVEF). In clinical practice, LVEF declines in patients with advanced cardiorespiratory disease are often much larger than 1%. Each 1-percentage-point increase in LVEF was associated with a 3.5% reduction in the hazard of death (HR, 0.965). Accordingly, a 5- or 10-percentage-point decrease in LVEF corresponds to an approximately 1.20-fold or 1.43-fold increase in the hazard of death, respectively. Thus, even small shifts in LVEF within the normal or high-normal range (e.g., from 65% to 55%) have profound prognostic implications for individual risk stratification under conditions of severe right heart overload, where preload is restricted. The high overlap in confidence intervals shown in the Kaplan–Meier curves reflects the clinical heterogeneity of ILD-PH, indicating that while no single parameter should guide clinical decisions in isolation, LVEF represents a crucial, universal marker of ventricular interdependence that must be monitored in tandem with other clinical indices. Therefore, rather than focusing on a 1% threshold for clinical decision-making, LVEF should be interpreted as a continuous risk gradient, where even modest serial declines (e.g., 5% to 10%) signify a substantial escalation in mortality risk, serving as a warning sign of failing left ventricular preload reserve.

This result broadcasts a clear message to clinicians: “Even in ILD patients with pulmonary hyperinflation or right heart enlargement—where advanced diastolic function assessment is difficult—clinicians must not overlook a decline in the universal parameter LVEF. Routinely monitoring this value is extremely useful for the risk stratification of high-risk patients”.

### 4.3. Importance of BMI as an Indicator of Systemic Status (Pulmonary Cachexia and Sarcopenia)

In addition to echocardiographic hemodynamic indices, the finding that a low baseline BMI was an independent poor prognostic factor strongly reinforces the systemic and consumptive nature of ILD. Chronic respiratory failure and severe PH trigger an increase in resting energy expenditure and the continuous secretion of systemic inflammatory cytokines such as TNF-α and IL-6. This precipitates a rapid decline in skeletal muscle mass (sarcopenia) and body weight (pulmonary cachexia), forging a vicious cycle of diminished exercise capacity and respiratory muscle fatigue [3,30].

A low BMI does not merely indicate poor nutritional status, but acts as a powerful surrogate marker denoting high disease activity and highly advanced decompensation of right heart failure. Maintaining LVEF and preserving nutritional status (BMI) can be likened to the “engine output” and the “chassis robustness” required to survive the intractable pathology of ILD-PH. Clinicians must not only track echocardiographic values but also detect weight trajectories and nutritional decline early, integrating comprehensive respiratory rehabilitation and nutritional interventions in tandem.

### 4.4. Limitations

This study has several limitations that should be acknowledged. First, because it is a single-center retrospective observational study, it is difficult to completely eliminate unknown confounding factors and selection bias. Second, pulmonary hypertension was not confirmed by the diagnostic gold standard, right heart catheterization (RHC), in all cases. Consequently, estimated RVSP via echocardiography represents an indirect surrogate marker and carries a risk of underestimation or overestimation in cases with poor tricuspid regurgitation signals or poor echo windows due to severe emphysema [7,15,31]. Third, the evaluation of LVEF and other echocardiographic indices relies to some extent on the operator’s proficiency. Although intra-observer and inter-observer variabilities were not formally measured in this cohort, all scans were performed by highly experienced sonographers and verified by board-certified cardiologists in a standardized laboratory, minimizing potential measurement bias. Nevertheless, the lack of formal reproducibility data is a methodological limitation, and future prospective trials should incorporate standardized variability assessments, especially when evaluating LVEF within normal ranges. Fourth, detailed information regarding patient treatments, including oxygen therapy, antifibrotic agents (e.g., pirfenidone, nintedanib), immunosuppressive therapies, pulmonary vasodilators, and rehabilitation programs, was not systematically collected in this database. While these therapies could have potentially influenced patient prognosis, their impact could not be adjusted for in our multivariable models. This lack of systematic treatment records is a common limitation of retrospective registry studies and should be taken into account when interpreting our findings. Fifth, the cohort in this study comprises patients registered between 2012 and 2017, prior to the widespread clinical use of inhaled treprostinil—a specific therapeutic agent for ILD-PH whose efficacy was proven by the INCREASE trial [32]. Consequently, the prognosis demonstrated in this study reflects the natural history under the standard of care of that era; in modern cohorts where specific pulmonary vasodilators are introduced, survival and dependency on left heart function may have altered [33]. The recruitment of patients was restricted to this historical window due to database constraints, and while updating the database to a modern cohort would be of interest, it represents a separate clinical undertaking. Nonetheless, this cohort provides a valuable baseline representing the natural progression of ILD-PH under standard clinical management of that period [34]. Nevertheless, the external validity of this study is exceptionally high precisely because it utilizes real-world clinical data where exhaustive RHC is impractical, thereby in no way diminishing the value of echocardiography as a routine screening tool. Future prospective multicenter cohort studies are desired to verify how the longitudinal trajectories of these echocardiographic parameters contribute to long-term prognosis. Sixth, myocardial strain analysis was not available in this study. Both left ventricular and right ventricular strain parameters have been reported to provide more sensitive assessments of myocardial dysfunction and may offer greater prognostic value than conventional echocardiographic indices. Therefore, the absence of strain measurements may have limited the comprehensive evaluation of subclinical biventricular dysfunction and its association with long-term outcomes [35]. Seventh, right ventricular–pulmonary arterial coupling was not assessed using indices such as the TAPSE/PASP ratio or the right ventricular free-wall strain/PASP ratio. These indices reflect the ability of the right ventricle to adapt to increased pulmonary arterial load and have been shown to provide important prognostic information in patients with pulmonary hypertension. Accordingly, the lack of RV–PA coupling assessment may have limited the characterization of right ventricular adaptation to pulmonary vascular load and its relationship with prognosis in this cohort.

## 5. Conclusions

In patients with pulmonary hypertension associated with interstitial lung disease (ILD-PH), reduced left ventricular diastolic function (Average *e’*) is a strong prognostic predictor in the overall cohort. Meanwhile, estimated right ventricular systolic pressure (RVSP) and patient’s low BMI, although associated with a poor prognosis in univariate analysis, showed only borderline trends in multivariable analysis and did not become independent significant predictors. However, a series of analyses revealed that under the severe condition of advanced right heart overload, the left ventricular pump function (LVEF) acts as a critical final bulwark determining patient survival. In routine clinical practice, in addition to monitoring the trends of RVSP and BMI, measuring the simple and universal parameter LVEF significantly contributes to the early risk stratification and the determination of optimal therapeutic strategies for high-risk patients with complex hemodynamics.

## Figures and Tables

**Table 1:** Baseline Characteristics (N = 415).

Parameter	Overall (*n* = 415)	IPF (*n* = 207)	Non-IPF (*n* = 208)
Age (years)	69.3 ± 9.4	71.5 ± 7.8	67.1 ± 10.2
Male Sex, *n* (%)	250 (60.2%)	159 (76.8%)	91 (43.8%)
BMI (kg/m^2^)	23.0 ± 3.8	23.2 ± 3.9	22.7 ± 3.7
Follow-up (months)	27.2 [13.6–45.1]	25.1 [13.9–42.6]	28.3 [13.4–48.5]
**Right Heart Parameters**			
TRV (m/s)	3.05 ± 0.60 (*n* = 26)	3.32 ± 0.66 (*n* = 8)	2.93 ± 0.56 *(n* = 18)
RVSP (mmHg)	36.72 ± 12.61 (*n* = 415)	38.35 ± 14.20 (*n* = 207)	35.09 ± 10.59 (*n* = 208)
TAPSE (mm)	21.30 ± 5.00 (*n* = 412)	20.73 ± 5.09 (*n* = 206)	21.87 ± 4.84 (*n* = 206)
RVFAC (%)	40.03 ± 10.71 (*n* = 414)	38.92 ± 10.28 (*n* = 207)	41.14 ± 11.04 (*n* = 207)
RV TEI Index	0.30 ± 0.18 (*n* = 309)	0.31 ± 0.18 (*n* = 158)	0.28 ± 0.17 (*n* = 151)
LV Eccentricity Index	0.99 ± 0.07 (*n* = 314)	0.98 ± 0.07 (*n* = 162)	0.99 ± 0.08 (*n* = 152)
**Left Heart Parameters**			
LADs (mm)	34.95 ± 6.41 (*n* = 415)	35.06 ± 6.09 (*n* = 207)	34.83 ± 6.73 (*n* = 208)
LA Volume Index (mL/m^2^)	32.28 ± 11.28 (*n* = 406)	32.47 ± 11.66 (*n* = 204)	32.09 ± 10.91 (*n* = 202)
LVEF (%)	64.73 ± 7.13 (*n* = 415)	64.40 ± 7.22 (*n* = 207)	65.05 ± 7.04 (*n* = 208)
Average *e’* (cm/s)	7.11 ± 1.91 (*n* = 415)	6.79 ± 1.76 (*n* = 207)	7.43 ± 2.00 (*n* = 208)
Average E/*e’*	10.06 ± 3.35 (*n* = 409)	10.31 ± 3.64 (*n* = 206)	9.80 ± 3.00 (*n* = 203)
IVSd (mm)	9.47 ± 1.61 (*n* = 415)	9.65 ± 1.47 (*n* = 207)	9.29 ± 1.73 (*n* = 208)
LVIDd (mm)	42.63 ± 4.92 (*n* = 415)	42.95 ± 4.89 (*n* = 207)	42.31 ± 4.94 (*n* = 208)
LVIDs (mm)	27.60 ± 4.16 (*n* = 415)	27.91 ± 4.30 (*n* = 207)	27.28 ± 4.00 (*n* = 208)
LVPWd (mm)	9.54 ± 1.58 (*n* = 415)	9.68 ± 1.55 (*n* = 207)	9.39 ± 1.60 (*n* = 208)

Abbreviations: BMI, body mass index; IPF, idiopathic pulmonary fibrosis; RVSP, right ventricular systolic pressure; LVEF, left ventricular ejection fraction. Continuous variables are expressed as mean ± standard deviation, and categorical variables as number (percentage).

**Table 2:** Multivariate Cox Proportional Hazards Analysis for All-Cause Mortality (Overall Cohort N = 415).

Variable	Hazard Ratio (HR)	95% CI	*p*-Value
Average *e’* (cm/s)	0.861	0.778–0.953	0.0038
Sex (Male)	1.652	1.117–2.445	0.0123
Age (years)	1.025	1.004–1.047	0.0225
BMI	0.950	0.903–1.000	0.0504
RVSP (mmHg)	1.013	1.000–1.026	0.0515
LVEF (%)	0.987	0.962–1.011	0.2800

Abbreviations: HR, hazard ratio; CI, confidence interval; BMI, body mass index; RVSP, right ventricular systolic pressure; LVEF, left ventricular ejection fraction. The model was adjusted for all variables listed in the table.

**Table 3:** Multivariate Cox Proportional Hazards Analysis Stratified by IPF Diagnosis.

Variable	IPF Subgroup (*n* = 207)			Non-IPF Subgroup (*n* = 208)		
	HR	95% CI	*p*-Value	HR	95% CI	*p*-Value
LVEF (%)	0.969	0.940–0.998	0.0305	1.021	0.973–1.071	0.3998
Average *e’* (cm/s)	0.854	0.748–0.974	0.0199	0.945	0.801–1.115	0.5038
BMI	0.943	0.885–1.005	0.0701	0.954	0.871–1.044	0.3016
Age (years)	1.021	0.991–1.053	0.1719	1.013	0.980–1.047	0.4373
RVSP (mmHg)	1.007	0.993–1.021	0.3365	1.021	0.990–1.053	0.1856
Sex (Male)	1.304	0.748–2.274	0.3452	1.352	0.697–2.626	0.3711

Abbreviations: HR, hazard ratio; CI, confidence interval; IPF, idiopathic pulmonary fibrosis; LVEF, left ventricular ejection fraction; BMI, body mass index; RVSP, right ventricular systolic pressure.

**Table 4:** Multivariate Cox Proportional Hazards Analysis in IPF Patients with High RVSP (≥35.7 mmHg, N = 104).

Variable	Hazard Ratio (HR)	95% CI	*p*-Value
LVEF (%)	0.965	0.936–0.995	0.0229
BMI	0.937	0.864–1.016	0.1167
Age (years)	1.029	0.987–1.072	0.1784
Average *e’* (cm/s)	0.932	0.775–1.122	0.4577
Sex (Male)	1.143	0.540–2.418	0.7264
RVSP (mmHg)	1.001	0.980–1.022	0.9531

Abbreviations: HR, hazard ratio; CI, confidence interval; IPF, idiopathic pulmonary fibrosis; RVSP, right ventricular systolic pressure; LVEF, left ventricular ejection fraction.

## Data Availability

The data presented in this study are available on request from the corresponding author due to privacy/ethical restrictions.

## Abbreviations

BMI	Body Mass Index
CI	Confidence Interval
HR	Hazard Ratio
ILD	Interstitial Lung Disease
IPF	Idiopathic Pulmonary Fibrosis
LV	Left Ventricular
LVEF	Left Ventricular Ejection Fraction
PH	Pulmonary Hypertension
RV	Right Ventricular
RVSP	Right Ventricular Systolic Pressure
